# Health financing in Brazil and the goals of the 2030 Agenda: high risk of failure

**DOI:** 10.11606/s1518-8787.2020054002414

**Published:** 2020-11-27

**Authors:** Fabiola Sulpino Vieira

**Affiliations:** Instituto de Pesquisa Econômica Aplicada Diretoria de Estudos e Políticas Sociais BrasíliaDF Brasil Instituto de Pesquisa Econômica Aplicada. Diretoria de Estudos e Políticas Sociais. Brasília, DF, Brasil

**Keywords:** Unified Health System, Health Care Rationing, Public Health Policy, Health Expenditures, Sustainable Development

## Abstract

**OBJECTIVE:**

To examine the financing of the Unified Health System (SUS) from 2010 to 2019 and analyze the recent trends in the allocation of federal resources to large areas of operation of the system, as well as the possibility of achieving the Sustainable Development Goal (SDG) 3 of the 2030 Agenda.

**METHODS:**

Data from the budgetary and financial execution of the federation entities were obtained. Transfers from the Brazilian Ministry of Health (MH) to the municipal and state departments and their direct applications were identified according to large final areas of SUS and middle areas. Basic descriptive statistics, graphs and tables were used to analyze the execution of expenses by these areas.

**RESULTS:**

Public spending *per capita* on health increased between 2010 and 2018. However, compared to 2014, it reduced 3% in 2018. There was a displacement of the allocation of federal resources to the detriment of transfers to the states (−21%). There are also losses of health surveillance in favor of primary care and pharmaceuticals. In the case of primary care, the increase in spending was tied to changes in policy and the expansion of resources allocated by parliamentary amendments. In the case of pharmaceuticals, the increase was due to the incorporation of new drugs, including vaccines, judicialization, increased spending on blood products and centralization, in the MH, of the purchase of items of high budgetary impact.

**CONCLUSION:**

If there is no change in the current SUS financing framework, something unlikely under Constitutional Amendment No. 95, associated with the redefinition of health policy priorities, the risk of non-compliance with the SDG 3 of the Agenda 2030 is very high.

## INTRODUCTION

The 2030 Agenda is a declaration reflecting the commitment made by the 193 Member States of the United Nations (UN) in 2015 to achieve a set of 17 Sustainable Development Goals (SDG), which are based on 169 goals aimed at promoting prosperity and well-being of populations based on sustainability worldwide ^1^ .

For health, SDG 3 – Health and Well-being is composed of 13 global goals that were adapted to Brazilian reality and national priorities in 2018, especially because some of them had already been achieved by the country ^2^ . The adapted goals are a guide for the action of the Brazilian State, since they quantify specific objectives related to the reduction of infant mortality, universal access to health services, reduction of premature deaths from non-communicable diseases, among others, to be achieved by 2030.

Regarding financing, the 3c goal was adapted to “significantly increase health financing and the recruitment, development, training and retention of health personnel, especially in the most vulnerable territories.” The text of the overall goal regarding health financing was not changed, nor was an indicator defined to monitor the allocation of resources to the Unified Health System (SUS) ^2^ .

The contemporary political, economic and social context of the country should explain it. The economic crisis that began in 2014 strongly compromised the finances of federal entities, particularly the Union and states ^3^ , with impacts on the allocation of resources to health ^4^ . And, in the federal government, the response to the crisis came with the implementation of a fiscal austerity policy, resulting in a significant limitation to the expansion of SUS financing ^5^^,^^6^ and spending cuts of other public policies, with consequences on constitutionally registered rights ^7^ .

Financial resources are essential for the supply of health goods and services and, in this sense, the State plays a fundamental role in ensuring social protection for the entire population, defending individuals against significant financial losses and impoverishment due to the out-of-pocket payment for health services ^8^ .

Regarding SDG 3, the achievement of the goals assumed by Brazil depends on the increase in public financing, not only for the SUS, to expand access to and quality of health services, but also on other social policies that impact factors related to the population's living and working conditions – the social determinants of health.

Considering the still insufficient financing of SUS and the implementation of fiscal austerity measures ^3^^,^^9^ , uncertainties about advances in public health in the country in the next two decades increase. Thus, the objectives of this article are to examine the financing of the SUS from 2010 to 2019 and to analyze the recent trends in the allocation of federal resources to large areas of operation of the system, as well as the possibility of achieving the SDG 3 of the 2030 Agenda.

## METHODS

A study was conducted on the recent trajectory of SUS financing from the analysis of expenditures of the three spheres of government between 2010 and 2019.

Initially, to contextualize Brazil from an international perspective, the indicator of public expenditure on health *per capita* in 2017 from selected countries (amounts in dollars per parity of purchasing power – PPP) was obtained from the website of the Organization for Economic Cooperation and Development (OECD), which consists of government expenditure and/or compulsory health schemes ^10^ . This year was chosen due to the availability of comparable information more recent for Brazil ^11^ . The indicator value in current Reais (R$) was adjusted for PPP dollars by applying the correction index made available by the OECD for that year.

The expenditure by the three levels of government with public health actions and services were surveyed. These expenses are those considered for the purposes of analyzing the minimum application in health. They represent reserves of amounts made in the budget of each member of the Federation, in each fiscal year, to honor, later, actual expenses with the SUS.

Three public sources of data were used: i) the *Siga Brasil* system, for Union expenditure ^12^ ; ii) the *Sistema de Informações sobre Orçamentos Públicos em Saúde* (Siops – Information System on Public Health Budgets), for the expenditures of states, the Federal District and municipalities, in addition to data from population ^13^ ; and iii) the annual archives of transfer of resources from the National Health Fund (NHF) to state and municipal health funds ^14^ .

The *per capita* public expenditure on health was estimated for each level of government, based on the committed expenditure from 2010 to 2018, presenting it, in national currency, in 2018 values with monetary correction by the average *Índice de Preços ao Consumidor Amplo* (IPCA – Broad Consumer Price Index).

For the federal government, additionally, the expenses actually paid by application modality were identified to analyze its execution according to the levels of government, grouping these modalities into: i) transfers to the states and the Federal District; (ii) transfers to municipalities; iii) direct applications (from the Brazilian Ministry of Health – MH) and transfers abroad, and iv) other modalities. These amounts, which are actual disbursements made by the public administration, result from the sum of the expenses paid for the financial year and the remaining amount to be paid in that fiscal year. Remaining amount to be paid are expenses that were committed in previous years, but were not paid until December 31 of the year in which they were committed, and can be entered and re-entered for payment in subsequent financial years.

The use of the variable “expenditure effectively paid” for the expenditure of the MH is important for the joint analysis of the amounts directly executed by the federal government and those passed on by the NHF to municipal and state health funds by SUS large final areas, namely: primary care, pharmaceuticals, medium and high complexity care (MHC) and health surveillance; and to finance expenses with middle areas: management and investments.

NHF transfers by area of activity were identified from 2010 to 2017 by the six financing blocks as designated in the annual transfer files. For 2018 and 2019, the information about the block is not sufficient due to changes in the allocation of resources of the Ministry of Health by MH Ordinance No. 3,992/2017, which organized the transfers in two financing blocks. In these two years, the values for investments come from the “investments” block, and the other expenses are obtained from the opening of the “costing” block per group.

The total of expenses paid in the modalities direct application and transfers abroad from the federal government was also classified by the areas of operation of the SUS and middle areas mentioned. For such purpose, the data obtained from *Siga Brasil* for these two modalities were opened: by group of expenditure nature (GEN), to identify costs and investment expenses; by subfunction, to discriminate the area of expenditure; and per budget action, to analyze the consistency of the classification by subfunction and to expense relocation, if necessary. In this article, expenses with the acquisition of vaccines, other immunobiological drugs and supplies were relocated from epidemiological surveillance to pharmaceuticals, since immunobiological drugs represent a significant part of the budget and are medicines (actions 6031 and 20YE). Spending on budget action 8670, which financed epidemiological surveillance actions and services and also supplies between 2010 and 2012, remained classified as health surveillance. Expenses with indigenous health care were included in primary care, and expenses with research and technological development were classified into management.

After the necessary adjustments in the classification of expenses and the monetary update of the values by the application of the IPCA, correcting them to 2019 reais, basic descriptive statistics, graphs and tables were used to analyze the execution of expenses by large areas of SUS operation, according to its relationship with SDG 3 targets of the 2030 Agenda.

## RESULTS

**Figure 1:**
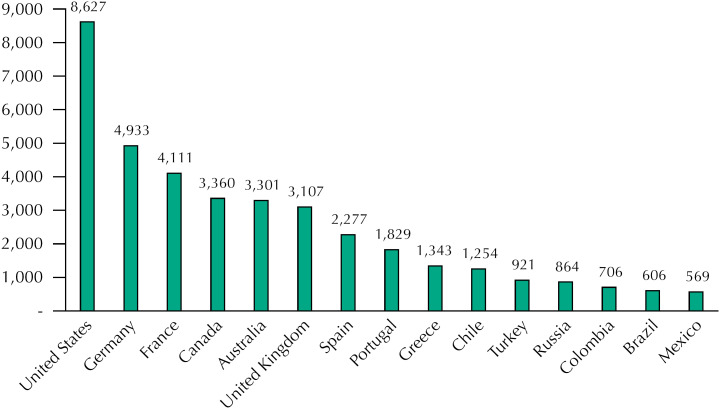
Public expenditure on health *per capita* of selected countries, in dollars for purchasing power parity (2017).

Source: Own elaboration based on data from the Brazilian Institute of Geography and Statistics (IBGE) ^11^ , for the *per capita* expenditure of the government in Brazil, and Organization for Economic Cooperation and Development (OECD) for *per capita* government expenditure and/or compulsory health financing schemes in other countries. In the case of Brazil, the *per capita* value was converted into dollars by purchasing power parity by the conversion index made available by the OECD.

Figure 1 shows the per capita public expenditure in 2017 of selected countries. The magnitude of the difference between Brazil's spending and that of countries that also have a universal health system is significant, being greater for that of the United Kingdom (which is 5 times the Brazilian expenditure), that of Spain (3.8 times) and that of Portugal (3 times). Brazil's spending is less than half of the expenditure of Chile, a country that has a limited public social protection system.

From the international comparison to a national federative view, the Table shows the evolution of expenditures committed *per capita* in Brazil by level of government between 2010 and 2018. Based on it, there is an increase in federal spending (8%), of the state – which includes that of the Federal District – (8%) and the municipal (23%) in the period analyzed. Considering the resources allocated by the three levels, *per capita* expenditure increased 13%, from R$ 1,165 in 2010 to R$ 1,311 in 2018, in constant values. However, in the most recent years, *per capita* expenditure reduced by 2% between 2014 and 2018 in the federal government, 5% in the states, 4% in the municipalities and 3% for the consolidated of the three levels of government. In absolute terms, committed Union expenditure increased 19%, from R$ 99.5 billion to R$ 122.6 billion, in constant amounts, between 2010 and 2019.

**Table:** Public expenditure per capita on health in Brazil by level of government (2010–2018).

Year	In 2018 Reals			
	Federal	State	Municipal	Brazil
2010	520	314	331	1,165
2011	566	327	362	1,255
2012	590	332	385	1,307
2013	558	351	403	1,312
2014	576	359	422	1,358
2015	571	345	412	1,328
2016	553	329	408	1,291
2017	573	342	412	1,327
2018	563	341	407	1,311

Source: *Siga Brasil* for expenditure committed data on public health actions and services (PHAS) from the federal level, and *Sistema de Informações sobre Orçamentos Públicos em Saúde* (Siops – Information System on Public Health Budgets) for data on expenditure committed to PHAS from the state and municipal levels and the population. The values were deflated by the *Índice de Preços ao Consumidor Amplo* (IPCA – Broad Consumer Price Index) for 2018.

**Figure 2:**
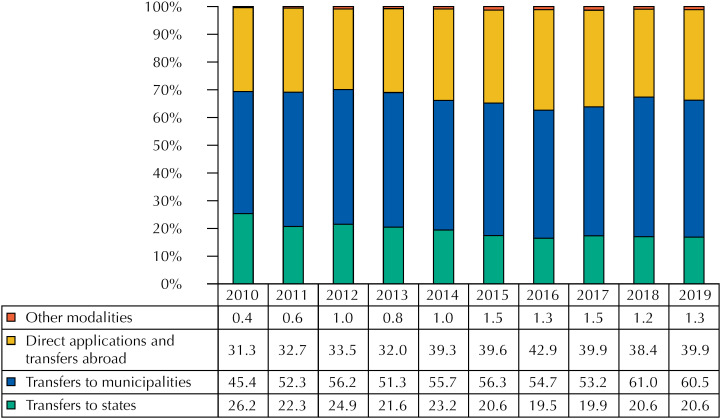
Federal government expenditure by application modality in billions of reais in 2019 (2010–2019).

Source: Own elaboration based on data from *Siga Brasil* on expenses paid in each fiscal year, plus the remains payable in the year.

Figure 2 shows details of the federal execution regarding the expenses actually paid by application modality, from which it is possible to verify a reduction in transfers from the Brazilian Ministry of Health to the state health departments (−21%), and an increase in direct applications plus transfers abroad (28%) and transfers to municipal health departments (33%) between 2010 and 2019. The amount of expenses actually paid increased 18%, from R$ 103.3 billion to R$ 122.4 billion in this period.

The values were deflated by the average *Índice de Preços ao Consumidor Amplo* (IPCA – Broad Consumer Price Index) for 2019.

**Figure 3:**
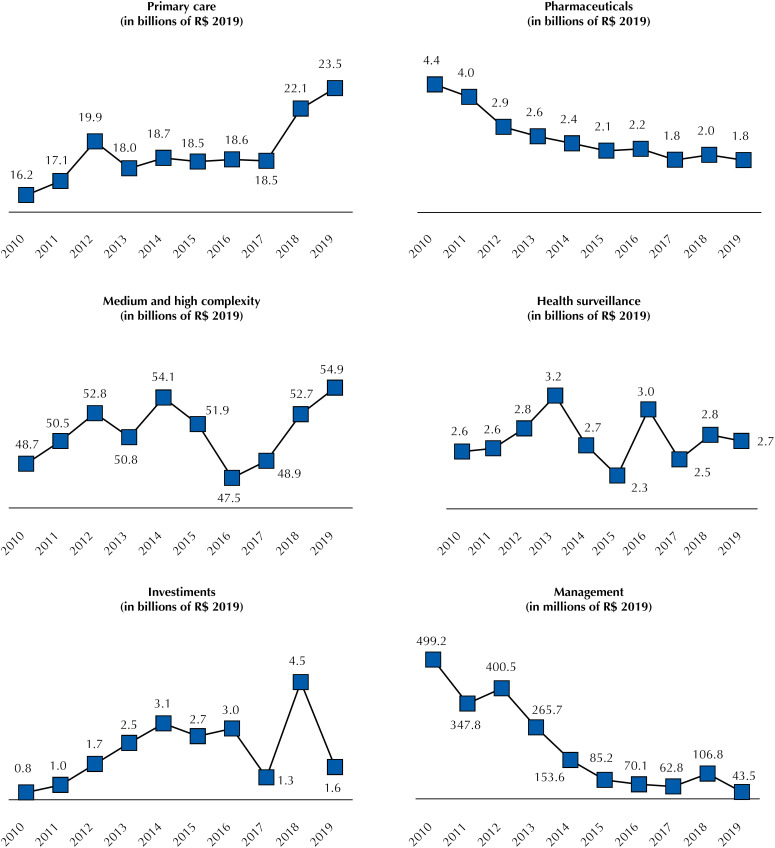
Transfers from the federal government to states and municipalities by health expenditure group (2010–2019).

Source: Own elaboration based on data from the National Health Fund.

Figure 3 shows that the expenses actually paid by the Ministry of Health, which were executed in the modalities of transfer to the states (including the Federal District) and to the municipalities, added – called by the NHF of transfers – are presented by SUS final areas of operation and by middle areas. Between 2010 and 2019, there was: i) an increase in transfers to primary care financing, especially from 2017 (46%, from R$ 16.2 billion to R$ 23.5 billion), MHC (13%, from R$ 48.7 billion to R$ 54.9 billion), health surveillance (4%, from R$ 2.6 billion to R$ 2.7 billion) and investments (116%, from R$ 0.8 billion to R$ 1.6 billion); and ii) reduction of transfers to pharmaceuticals (−59%, from R$ 4.4 billion to R$ 1.8 billion) and management (−91%, from R$ 499.2 million to R$ 43.5 million). A significant reduction in these transfers to MHC between 2014 and 2016 (−12%) and a peak in transfers to investments in 2018 (R$ 4.5 billion).

The values were deflated by the average *Índice de Preços ao Consumidor Amplo* (IPCA – Broad Consumer Price Index) for 2019.

**Figure 4:**
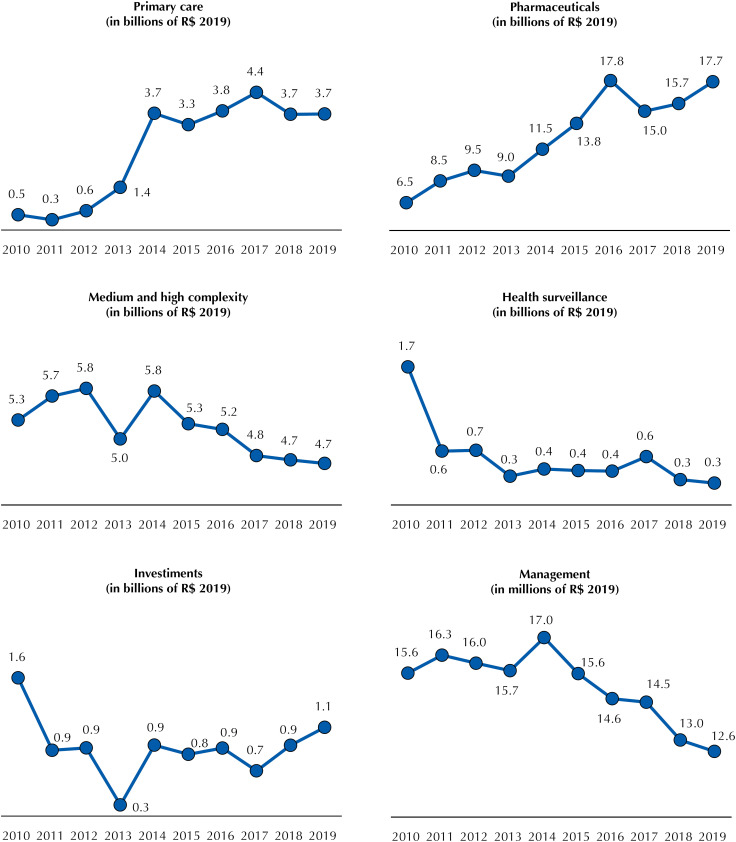
Direct applications and transfers abroad from the federal government by health expenditure group (2010–2019).

Source: Own elaboration based on data from *Siga Brasil* on expenses paid in each fiscal year, plus the remains payable in the year.

Figure 4 shows the expenses paid in the modalities of direct application and transfer abroad by the MH, added together, by the same areas of operation of the SUS and period. There was a significant increase in spending on primary care (664%, from R$ 0.5 billion to R$ 3.7 billion) and on pharmaceuticals (170%, from R$ 6.5 billion to R$ 17.7 billion), in parallel with the reduction of MHC expenses (−13%, R$ 5.3 billion to R$ 4.7 billion), health surveillance (−84%, from R$ 1.7 billion to R$ 0.3 billion), investments (−31%, from R$ 1.6 billion to R$ 1.1 billion) and management (−20% from R$ 15.7 billion to R$ 12.6 billion). The large fall in surveillance spending between 2010 and 2011 (−61%) stands out.

The values were deflated by the average *Índice de Preços ao Consumidor Amplo* (IPCA – Broad Consumer Price Index) for 2019.

## DISCUSSION

Since the promulgation of the 1988 Federal Constitution, SUS financing has been a constant concern for researchers and public managers committed to ensuring access to health goods and services in a universal, equal and integral way. The apprehension with the present and the future of the system is amplified in the face of the decisions of public agents, especially within the federal government, in a sense different from that expressed in the Constitution ^5^^,^^15^^–^^17^ .

Constitutional Amendment No. 95/2016 (EC 95) imposes an important restriction on SUS financing. Until recently, this measure was considered the great attack against the realization of the right to health in Brazil, and the threats became more serious with a possible elimination of the mandatory minimum expenditure on health by the levels of government ^4^ and with the Proposed Amendment to Constitution No. 188, whose objective is that the additional expenditure on health or education beyond the floor of each area can be discounted from the other, to verify the minimum applications ^18^ .

Figure 1 shows that Brazil's *per capita* public health expenditure has been reduced in recent years and is very low compared to that of countries with a universal health system. And the prospects are not good, because the Brazilian position in the international context tends to worsen under EC 95. There is already an increase in the participation of households in spending on health goods and services, from 53.5% in 2010 to 56.8% in 2017 ^11^ . Although the exact figures for Brazil are not available so far, a significant part of these expenses is out-of-pocket, especially for the purchase of medicines, and that the growth of this type of disbursement is one of the main factors of worsening equity in the financing of health systems ^19^ .

Considering the expenses paid by the Ministry of Health by modality of application, direct execution by the federal government and transfers to municipalities have been prioritized in recent years. Since the amount of these expenses executed increased by 19% between 2010 and 2019 and the applications in the aforementioned modalities increased 28% and 33%, respectively, there was a displacement of the allocation of resources to the detriment of transfers to the states, which decreased 21%. There are also losses in health surveillance in favor of primary care and pharmaceuticals.

Initiatives such as the *SUS Legal* , a proposal agreed in the Tripartite Intermanagers Commission so that the transfers by the Ministry of Health were totally delimited from specific applications ^20^ , and decisions such as increasing the participation of parliamentary amendments in the allocation of federal resources and centralizing some actions in the federal government help explain this displacement. Regarding the *SUS Legal* , under the strong influence of the municipalist agenda, greater financial flexibility was approved for the execution of the transfers from MH, but not the total disconnection intended ^20^ . Regarding parliamentary amendments, there was an increase in its participation in SUS costing, with an increase in transfers to the increase of the Primary Care Floor (PCF) and the MHC Ceiling ^21^ . These and other initiatives of the federal government in response to the economic crisis are pointed out as causes of the weakening of regional governance in the SUS, which has the states as important actors ^22^ , and may damage the integrality of health care.

Important issues emerge when the execution of the MH expenses by area of activity ( Figures 3 and 4 ) in parallel to the SFG 3 targets of the 2030 Agenda is analyzed. Regarding primary care, there is an expansion of resources executed directly by the Ministry of Health or by transfer abroad, particularly to the Pan American Health Organization – with a reduction in the amount in the last two years – and an expansion of transfers to the municipalities. In direct application, the initial increase occurred, especially since 2013, with the federal participation in the provision of professionals under the *Programa Mais Médicos* (More Doctors Program), and its fall is probably related to the departure of Cuban physicians in 2018 and the replacement of that program by *Médicos pelo Brasil* (Doctor throughout Brazil) in 2019. In transfers to municipalities, there was an increase in the resources passed on to increase the PCF and the MHC Ceiling in recent years by parliamentary amendments ^21^ .

At first, the focus on primary care is considered a crucial measure for the effectiveness of universal health systems. However, studies showing its effectiveness in Brazil evaluated the Family Health Strategy (FHS) model ^23^ . No robust studies were identified on the effects of the “traditional” model on the health of the population, in which care is done strictly in the basic health unit by the professionals available. The FHS began to compete for resources with the traditional model in consequence of recent measures of flexibilization of the models of teams financed by federal resources ^24^^,^^25^ . This situation can result in unintended consequences for the effectiveness of primary care in the country. A possible reduction in FHS coverage could increase the mortality rate due to primary care-sensitive conditions in 5.8% by 2030, and in 8.6% in case of closure of Mais Médicos ^26^ .

Transfers to MHC in the analyzed series had a smaller increase than that observed for primary care. Moreover, these transfers were most affected by the economic crisis and the contingencies of payment of expenses of the MH between 2014 and 2016. In MH direct application, the decrease in execution since 2014 can be partially explained by the decrease in resources passed on to university hospitals under the *Programa Nacional de Reestruturação dos Hospitais Universitários Federais* (National Program for Restructuring Federal University Hospitals), which was around R$ 630.3 million in 2014, in constant amounts, and without any transfer in 2019 for the payment of current expenses. MHC actions and services are fundamental to ensure comprehensive care in SUS, and these two levels of complexity are often pointed out as system bottlenecks. Thus, the low priority they have had in recent years is worrisome.

The reduction of transfers for pharmaceuticals is associated with the decision to centralize the purchase of drugs of the Specialized Component of Pharmaceutical Assistance in the Ministry of Health, previously under the responsibility of the states. This decision, together with the incorporation of new drugs under federal financing, including vaccines, and the expansion of spending on blood products and judicialization explain the significant growth of MH direct application ^27^ . Moreover, in the last three years, the drugs of the Strategic Component of Pharmaceutical Assistance (immunobiological, antiretroviral and blood products) have been gaining space in the federal expenditure on pharmaceutical products, to the detriment of the items offered in primary care.

Regarding health surveillance expenditure, the higher direct application of the Ministry of Health in 2010 than in 2011 resulted from the implementation of actions and services to stop the pandemic by the influenza virus. In 2011, the execution level was already low and reduced 59% compared to 2019. There was also a reduction in management expenses by direct application of the MH and transfers to projects related to this area. Concerning MH, the diminution occurred due to a decrease in expenses with active personnel, which should be associated with a reduction in the number of professionals, which may generate greater worsening of the labor force in the agency.

The situation of SUS financing in recent years and changes in structuring policies create uncertainties about the future of health in the country. Fiscal austerity policies implemented in response to economic crises have been pointed out as a determining factor in the worsening of the health situation of the population worldwide, especially among the most socioeconomically vulnerable groups ^28^^,^^29^ . In Brazil, the impact of these policies on SUS has generated concern about the possibility of setbacks in the supply of health goods and services ^30^ and the achievement: of the goals related to the control of non-communicable chronic diseases ^31^ ; of the SDG, in general ^32^ ; and of the SDG 3 related to tuberculosis ^33^ . The fact that the Federal Government is the Federation authority with the highest fiscal capacity makes it possible to expect greater effort by the federal government to finance SUS, since states and municipalities are already at the limit of their financial possibilities ^4^ . Thus, greater allocation of federal resources is necessary to Brazil achieve SDG 3.

Although the achievement of these goals depends on a set of actions and services offered within the entire health system, and also on other public policies that act on social determinants of health, it can be said that, in what depends on the SUS, these purposes are more associated with one or another finalistic area of the system. The following SDGs 3 goals are closely related to primary care, MHC and health surveillance: 3.1 – reduction of maternal mortality; 3.2 – reduction of neonatal mortality; 3.3 – an end to aids, tuberculosis, malaria and tropical disease epidemics; 3.4 – reduction of premature mortality from non-communicable diseases, with the promotion of mental health, workers' health and suicide prevention; 3.5 – strengthening prevention and treatment due to the use of substances (narcotic drugs and alcohol abuse); 3.6 – reduction of deaths and injuries from traffic accidents, and 3.9 – substantial reduction of deaths and diseases from hazardous chemicals.

The following goals strongly involve the areas of primary care, MHC and pharmaceuticals: 3.7 – ensuring universal access to sexual and reproductive health services and supplies; and 3.8 – ensuring access to quality essential health services at all levels of care, and medicines and vaccines incorporated into the SUS. These goals are associated with health surveillance and system management: 3a – strengthening the implementation of the *Convenção-Quadro para o Controle do Tabaco no Brasil* (Framework Convention on Tobacco Control in Brazil), and 3d – capacity building for early warning, reduction and management of national and global health emergencies and risks. Goal 3b – support for research and development of health technologies and innovations –, in turn, is associated with management and investments in the SUS, whereas target 3c – a substantial increase in health financing and human resources development – involves fiscal policy decision that depends heavily on the heads of the executive branch in the three levels of government, in the first case, and actions within the management of the SUS, in the second.

In conclusion, in the case of financing, there has been a decrease in *per capita* spending in recent years, and the prospects are for a reduction of this indicator under the validity of EC 95, by freezing the federal minimum application in the SUS and by the delay that it causes in economy recovery, with consequences for the collection of states and municipalities and, therefore, for the allocation of resources by these entities to health ^5^^,^^6^ . Without sufficient funds for SUS financing and for other policies that act on social determinants of health, associated with the redefinition of health policy priorities, the achievement of SDG 3 goals is compromised, since it depends on the expansion of access to health goods and services and on improving the quality of the provision of these services. If there is no change in the current framework, the risk of non-compliance with these targets is very high.
