# Supplementary material for: Health financing in Brazil and the goals of the 2030 Agenda: high risk of failure
Source: Rev Saude Publica. 2020 Nov 27;54:127. doi: 10.11606/s1518-8787.2020054002414 (PMC7703550; doi:10.11606/s1518-8787.2020054002414)
Supplement: Translation

# Translation: O financiamento da saúde no Brasil e as metas da Agenda 2030: alto risco de insucesso

**Authors:** Fabiola Sulpino Vieira

**Affiliations:** BrasíliaDF Brasil Instituto de Pesquisa Econômica Aplicada. Diretoria de Estudos e Políticas Sociais. Brasília, DF, Brasil

**Keywords:** Sistema Único de Saúde, Alocação de Recursos para a Atenção à Saúde, Políticas Públicas de Saúde, Gastos em Saúde, Desenvolvimento Sustentável

## Abstract

**OBJETIVO:**

Examinar o financiamento do Sistema Único de Saúde (SUS) de 2010 a 2019 e analisar as tendências recentes da alocação de recursos federais para grandes áreas de atuação do sistema, bem como a possibilidade de alcance das metas do Objetivo de Desenvolvimento Sustentável (ODS) 3 da Agenda 2030.

**MÉTODOS:**

Dados da execução orçamentário-financeira dos entes da Federação foram obtidos. As transferências do Ministério da Saúde (MS) para as secretarias municipais e estaduais e suas aplicações diretas foram identificadas segundo grandes áreas finalísticas do SUS e áreas-meio. Estatísticas descritivas básicas, gráficos e tabelas foram utilizados para a análise da execução das despesas por essas áreas.

**RESULTADOS:**

O gasto público per capita com saúde aumentou entre 2010 e 2018. Contudo, em relação a 2014, teve redução de 3% em 2018. Constatou-se deslocamento da alocação de recursos federais em prejuízo das transferências aos estados (−21%). Também se observaram perdas da vigilância em saúde em favor da atenção básica e da assistência farmacêutica. No caso da atenção básica, o aumento do gasto veio atrelado a mudanças na política e à ampliação dos recursos alocados por emendas parlamentares. Já no caso da assistência farmacêutica, o aumento se deu pela incorporação de novos medicamentos, incluindo vacinas, pela judicialização, pelo aumento dos gastos com hemoderivados e pela centralização, no MS, da compra de itens de alto impacto orçamentário.

**CONCLUSÃO:**

Caso não haja mudança no quadro atual de financiamento do SUS, algo improvável sob a vigência da Emenda Constitucional nº 95, associada à redefinição das prioridades das políticas de saúde, o risco de não cumprimento das metas do ODS 3 da Agenda 2030 é muito alto.

## INTRODUÇÃO

A Agenda 2030 é uma declaração que traduz o compromisso assumido pelos 193 Estados-membros da Organização das Nações Unidas (ONU), em 2015, para o alcance de um conjunto de 17 Objetivos de Desenvolvimento Sustentável (ODS), que se desdobram em 169 metas, visando à promoção da prosperidade e do bem-estar das populações de forma sustentável em todo o mundo ^1^ .

Especificamente para o campo da saúde, o ODS 3 – Saúde e Bem-Estar é composto por 13 metas globais que, no caso do Brasil, foram adequadas à realidade e às prioridades nacionais em 2018, especialmente porque algumas delas já haviam sido atingidas pelo país ^2^ . As metas readequadas são um guia para a ação do Estado brasileiro por quantificarem objetivos específicos relacionados à redução da mortalidade infantil, ao acesso universal a serviços de saúde, à diminuição das mortes prematuras por doenças não transmissíveis, entre outros, a serem alcançados até 2030.

No tocante ao financiamento, a meta 3c foi readequada para “aumentar substancialmente o financiamento da saúde e o recrutamento, desenvolvimento, formação e retenção do pessoal de saúde, especialmente nos territórios mais vulneráveis”. Nenhuma alteração da redação da meta global foi feita em relação ao financiamento da saúde, tampouco foi definido indicador para acompanhar a alocação de recursos para o Sistema Único de Saúde (SUS) ^2^ .

Explicação para este fato pode ser encontrada no contexto político, econômico e social contemporâneo do país. A crise econômica iniciada em 2014 comprometeu fortemente as finanças dos entes federados, particularmente da União e dos estados ^3^ , com impactos sobre a destinação de recursos para a saúde ^4^ . E, no governo federal, a resposta à crise veio com a implantação de política de austeridade fiscal, resultando em expressiva limitação à ampliação do financiamento do SUS ^5^^,^^6^ e em cortes de gastos com outras políticas públicas, com consequências sobre direitos constitucionalmente inscritos ^7^ .

Recursos financeiros são insumos essenciais para a oferta de bens e serviços de saúde e, nesse sentido, o Estado tem papel fundamental na garantia de proteção social para toda a população, defendendo os indivíduos contra perdas financeiras significativas e empobrecimento em virtude do pagamento por serviços de saúde diretamente do bolso ( *out-of-pocket* ) ^8^ .

No tocante ao ODS 3, o alcance das metas assumidas pelo Brasil depende do aumento do financiamento público, não apenas para o SUS, a fim de ampliar o acesso e a qualidade dos serviços de saúde, mas também para outras políticas sociais que impactam fatores relacionados às condições de vida e de trabalho da população – os determinantes sociais da saúde.

Considerando o ainda insuficiente financiamento do SUS e a implantação de medidas de austeridade fiscal ^3^^,^^9^ , aumentam as incertezas sobre os avanços em saúde pública no país nas próximas duas décadas. Assim, os objetivos deste artigo são examinar o financiamento do SUS de 2010 a 2019 e analisar as tendências recentes da alocação de recursos federais para grandes áreas de atuação do sistema, bem como a possibilidade de alcance das metas do ODS 3 da Agenda 2030.

## MÉTODOS

Realizou-se estudo sobre a trajetória recente do financiamento do SUS a partir da análise dos gastos das três esferas de governo entre 2010 e 2019.

Inicialmente, para contextualizar o Brasil em perspectiva internacional, obteve-se do sítio eletrônico da Organização para a Cooperação e o Desenvolvimento Econômico (OCDE) o indicador de despesa pública com saúde per capita em 2017 de países selecionados (valores em dólares por paridade do poder de compra – PPC), que consiste nas despesas do governo e/ou de esquemas compulsórios de saúde ^10^ . Esse ano foi escolhido em função da disponibilidade de informação comparável mais recente para o Brasil ^11^ . O valor do indicador em reais (R$) correntes foi ajustado para dólares PPC pela aplicação do índice de correção disponibilizado pela OCDE para aquele ano.

As despesas empenhadas pelas três esferas de governo com ações e serviços públicos de saúde foram levantadas. Essas despesas são as consideradas para fins de apuração da aplicação mínima em saúde. Elas representam reservas de valores feitas no orçamento de cada ente da Federação, em cada exercício, para honrar, posteriormente, gastos efetivos com o SUS.

Três fontes públicas de dados foram utilizadas: i) o sistema Siga Brasil, para as despesas da União ^12^ ; ii) o Sistema de Informações sobre Orçamentos Públicos em Saúde (Siops), para as despesas dos estados, do DF e dos municípios, além de dados da população ^13^ ; e iii) os arquivos anuais de repasse de recursos do Fundo Nacional de Saúde (FNS) para os fundos de saúde estaduais e municipais ^14^ .

Calculou-se para cada esfera de governo o gasto público per capita com saúde, com base na despesa empenhada do período de 2010 a 2018, apresentando-o, em moeda nacional, em valores de 2018 com correção monetária pelo Índice de Preços ao Consumidor Amplo (IPCA) médio.

Para o governo federal, adicionalmente, identificaram-se as despesas efetivamente pagas por modalidade de aplicação, a fim de analisar a sua execução segundo as esferas de governo, agrupando-se tais modalidades em: i) transferências aos estados e ao DF; ii) transferências a municípios; iii) aplicações diretas (do Ministério da Saúde – MS) e transferências ao exterior; e iv) outras modalidades. Esses valores, que são desembolsos efetivos feitos pela administração pública, são resultantes da soma das despesas pagas do exercício financeiro e dos restos a pagar pagos nesse exercício. Restos a pagar são despesas que foram empenhadas em exercícios anteriores, mas não foram pagas até o dia 31 de dezembro do ano em que foram empenhadas, podendo ser inscritas e reinscritas para pagamento em exercícios financeiros subsequentes.

O uso da variável “despesa efetivamente paga” para o gasto do MS é importante para a análise conjunta dos valores executados diretamente pelo governo federal e daqueles repassados pelo FNS aos fundos municipais e estaduais de saúde por grandes áreas finalísticas de atuação do SUS, quais sejam: atenção básica, assistência farmacêutica, atenção de média e alta complexidade (MAC) e vigilância em saúde; e para financiar despesas com áreas-meio: gestão e investimentos.

As transferências do FNS por área de atuação foram identificadas no período de 2010 a 2017 pelos seis blocos de financiamento assim designados nos arquivos de repasse anuais. Para 2018 e 2019, a informação sobre o bloco não é suficiente em razão das mudanças na alocação de recursos do MS por meio da Portaria GM/MS nº 3.992/2017, que organizou os repasses em dois blocos de financiamento. Nesses dois anos, os valores para investimentos são provenientes do bloco “investimentos”, e as demais despesas são obtidas a partir da abertura do bloco “custeio” por grupo.

A soma das despesas pagas nas modalidades aplicação direta e transferências ao exterior do governo federal também foi classificada pelas áreas de atuação do SUS e áreas-meio mencionadas. Para tanto, os dados obtidos do Siga Brasil para essas duas modalidades foram abertos: por grupo de natureza de despesa (GND), para identificar despesas de custeio e de investimento; por subfunção, para discriminar a área do gasto; e por ação orçamentária, para análise da consistência da classificação por subfunção e para realocação de despesas, caso fosse necessário. Neste artigo, despesas com a aquisição de vacinas, outros imunobiológicos e insumos foram realocadas de vigilância epidemiológica para assistência farmacêutica, uma vez que imunobiológicos têm grande peso no orçamento e são medicamentos (ações 6031 e 20YE). Gastos com a ação orçamentária 8670, que financiou ações e serviços de vigilância epidemiológica e também insumos entre 2010 e 2012, permaneceram classificados como vigilância em saúde. Despesas com atenção à saúde indígena foram incluídas em atenção básica, e despesas com pesquisa e desenvolvimento tecnológico foram registradas em gestão.

Após os ajustes necessários na classificação das despesas e a atualização monetária dos valores pela aplicação do IPCA, corrigindo-os para reais de 2019, estatísticas descritivas básicas, gráficos e tabelas foram utilizados para a análise da execução das despesas por grandes áreas de atuação do SUS, segundo sua relação com as metas do ODS 3 da Agenda 2030.

## RESULTADOS

Na Figura 1 , apresenta-se o gasto público per capita em 2017 de países selecionados. A magnitude da diferença entre o gasto do Brasil e o de países que também possuem sistema universal de saúde é expressiva, sendo maior para o do Reino Unido (que é de 5 vezes o gasto brasileiro), o da Espanha (3,8 vezes) e o de Portugal (3 vezes). O gasto do Brasil chega a ser menos que a metade do gasto do Chile, país este que tem um sistema de proteção social público limitado.

**Figura 1:** Gasto público em saúde per capita de países selecionados, em dólares por paridade do poder de compra (2017).

Fonte: Elaboração própria a partir de dados do Instituto Brasileiro de Geografia e Estatística ^11^ , para a despesa per capita do governo no Brasil, e Organização para a Cooperação e o Desenvolvimento Econômico (OCDE) para a despesa per capita do governo e/ou de esquemas compulsórios de financiamento da saúde dos demais países. No caso do Brasil, o valor per capita foi convertido em dólares por paridade do poder de compra pelo índice de conversão disponibilizado pela OCDE.

Saindo da comparação internacional para uma visão federativa nacional, apresenta-se na Tabela a evolução das despesas empenhadas per capita no Brasil por esfera de governo entre 2010 e 2018. A partir dela, observa-se aumento do gasto federal (8%), do estadual – que inclui o do Distrito Federal – (8%) e do municipal (23%) no período analisado. Considerando os recursos alocados pelas três esferas, o gasto per capita cresceu 13%, passando de R$ 1.165, em 2010, para R$ 1.311 em 2018, em valores constantes. Entretanto, quando o olhar se volta para os anos mais recentes, verifica-se redução da despesa per capita entre 2014 e 2018 em 2% no governo federal, 5% nos estados, 4% nos municípios e 3% para o consolidado das três esferas de governo. Em termos absolutos, as despesas da União empenhadas aumentaram 19%, passando de R$ 99,5 bilhões para R$ 122,6 bilhões, em valores constantes, entre 2010 e 2019.

**Tabela:** Gasto público per capita com saúde no Brasil por esfera de governo (2010–2018).

Ano	Em reais de 2018			
	Federal	Estadual	Municipal	Brasil
2010	520	314	331	1.165
2011	566	327	362	1.255
2012	590	332	385	1.307
2013	558	351	403	1.312
2014	576	359	422	1.358
2015	571	345	412	1.328
2016	553	329	408	1.291
2017	573	342	412	1.327
2018	563	341	407	1.311

Fonte: Siga Brasil para dados de despesa empenhada com ações e serviços públicos de saúde (ASPS) da esfera federal, e Sistema de Informações sobre Orçamentos Públicos em Saúde (Siops) para dados de despesa empenhada com ASPS das esferas estadual e municipal e da população. Os valores foram deflacionados pelo Índice de Preços ao Consumidor Amplo médio para reais de 2018.

Detalhamento da execução federal quanto às despesas efetivamente pagas por modalidade de aplicação é apresentado na Figura 2 , a partir da qual é possível verificar redução das transferências do MS às secretarias estaduais de saúde (−21%), e aumento das aplicações diretas acrescidas das transferências ao exterior (28%) e das transferências às secretarias municipais de saúde (33%) entre 2010 e 2019. O montante das despesas efetivamente pagas passou de R$ 103,3 bilhões para R$ 122,4 bilhões nesse período, perfazendo um aumento de 18%.

**Figura 2:** Despesas do governo federal com saúde por modalidade de aplicação em bilhões de reais de 2019 (2010–2019).

Fonte: Elaboração própria a partir de dados do Siga Brasil sobre despesas pagas em cada exercício, acrescidas dos restos a pagar no exercício.

Os valores foram deflacionados pelo Índice de Preços ao Consumidor Amplo médio para reais de 2019.

Na Figura 3 , as despesas efetivamente pagas pelo MS, que foram executadas nas modalidades de transferência aos estados (incluindo o Distrito Federal) e aos municípios, somadas – chamadas pelo FNS de repasses –, são apresentadas por áreas finalísticas de atuação do SUS e por áreas-meio. Entre 2010 e 2019, constata-se: i) aumento dos repasses para financiamento da atenção básica, especialmente a partir de 2017 (46%, de R$ 16,2 bilhões para R$ 23,5 bilhões), da MAC (13%, de R$ 48,7 bilhões para R$ 54,9 bilhões), da vigilância em saúde (4%, de R$ 2,6 bilhões para R$ 2,7 bilhões) e de investimentos (116%, de R$ 0,8 bilhão para R$ 1,6 bilhão); e ii) redução dos repasses para assistência farmacêutica (−59%, de R$ 4,4 bilhões para R$ 1,8 bilhão) e gestão (−91%, de R$ 499,2 milhões para R$ 43,5 milhões). Chama a atenção uma importante redução desses repasses para MAC entre 2014 e 2016 (−12%) e um pico nos repasses para investimentos em 2018 (R$ 4,5 bilhões).

**Figura 3:** Repasses do governo federal para estados e municípios por grupo de despesa em saúde (2010–2019).

Fonte: Elaboração própria a partir de dados do Fundo Nacional de Saúde.

Os valores foram deflacionados pelo Índice de Preços ao Consumidor Amplo médio para reais de 2019.

As despesas pagas nas modalidades de aplicação direta e transferência ao exterior do MS, somadas, são apresentadas pelas mesmas áreas de atuação do SUS e período na Figura 4 . Nota-se aumento expressivo dos gastos com atenção básica (664%, de R$ 0,5 bilhão para R$ 3,7 bilhões) e assistência farmacêutica (170%, de R$ 6,5 bilhões para R$ 17,7 bilhões), paralelamente à redução de despesas com MAC (−13%, de R$ 5,3 bilhões para R$ 4,7 bilhões), vigilância em saúde (−84%, de R$ 1,7 bilhão para R$ 0,3 bilhão), investimentos (−31%, de R$ 1,6 bilhão para R$ 1,1 bilhão) e gestão (−20%, de R$ 15,7 bilhões para R$ 12,6 bilhões). Destaca-se a grande queda das despesas com vigilância entre 2010 e 2011 (−61%).

**Figura 4:** Aplicações diretas e transferências ao exterior do governo federal por grupo de despesa em saúde (2010–2019).

Fonte: Elaboração própria a partir de dados do Siga Brasil sobre despesas pagas do exercício, acrescidas dos restos a pagar no exercício.

Os valores foram deflacionados pelo Índice de Preços ao Consumidor Amplo médio para reais de 2019.

## DISCUSSÃO

Desde a promulgação da Constituição Federal de 1988, o financiamento do SUS tem sido preocupação constante para pesquisadores e gestores públicos comprometidos com a garantia de acesso a bens e a serviços de saúde de forma universal, igualitária e integral. A apreensão com o presente e o futuro do sistema é amplificada diante das decisões de agentes públicos, especialmente no âmbito do governo federal, em sentido diverso daquele expresso na Constituição ^5^^,^^15^^–^^17^ .

A Emenda Constitucional nº 95/2016 (EC 95) impõe restrição importante ao financiamento do SUS. Até recentemente, essa medida foi considerada a grande investida contra a efetivação do direito à saúde no Brasil, mas as ameaças se tornaram mais graves com uma possível eliminação da obrigatoriedade de gasto mínimo em saúde pelas esferas de governo ^4^ e com a Proposta de Emenda à Constituição nº 188, cujo objetivo é que o gasto adicional em saúde ou em educação para além do piso de cada área possa ser descontado da outra, para fins de apuração das aplicações mínimas ^18^ .

Como mostra a Figura 1 , o gasto público per capita em saúde do Brasil sofreu redução nos últimos anos e é muito baixo em comparação ao de países com sistema universal de saúde. E as perspectivas não são boas, pois a posição brasileira em contexto internacional tende a piorar sob a EC 95. Já se observa aumento da participação das famílias na despesa com o consumo final de bens e serviços de saúde, que passou de 53,5%, em 2010, para 56,8% em 2017 ^11^ . Ainda que não se disponha, até o momento, de números exatos para o Brasil, sabe-se que parte significativa dessas despesas ocorre por desembolso direto, especialmente para a compra de medicamentos, e que o crescimento desse tipo de desembolso é um dos principais fatores de piora da equidade no financiamento dos sistemas de saúde ^19^ .

Considerando as despesas pagas pelo MS por modalidade de aplicação, observa-se priorização da execução direta pelo governo federal e dos repasses para os municípios nos últimos anos. Como o montante executado dessas despesas aumentou 19% entre 2010 e 2019 e as aplicações nas modalidades mencionadas cresceram 28% e 33%, respectivamente, constata-se deslocamento da alocação de recursos em prejuízo das transferências aos estados, que tiveram queda de 21%. Também se observam perdas da vigilância em saúde em favor da atenção básica e da assistência farmacêutica.

Iniciativas como o SUS Legal, proposta pactuada na Comissão Intergestores Tripartite para que os repasses do MS fossem totalmente desvinculados de aplicações específicas ^20^ , e decisões como as de aumentar a participação das emendas parlamentares na alocação dos recursos federais e de centralizar algumas ações no governo federal ajudam a explicar esse deslocamento. Quanto ao SUS Legal, sob forte influência da agenda municipalista, aprovou-se maior flexibilidade financeira para a execução dos repasses do MS, mas não a desvinculação total pretendida ^20^ . Em relação às emendas parlamentares, houve aumento de sua participação no custeio do SUS, com ampliação das transferências para o incremento do Piso da Atenção Básica (PAB) e do Teto MAC ^21^ . Essas e outras iniciativas do governo federal em resposta à crise econômica são apontadas como causas da fragilização da governança regional no SUS, que tem os estados como um ator importante ^22^ , podendo ser prejudiciais à integralidade do cuidado da saúde.

Analisando-se a execução das despesas do MS por área de atuação ( Figuras 3 e 4 ) em paralelo às metas do ODS 3 da Agenda 2030, questões importantes emergem. No tocante à atenção básica, observa-se ampliação dos recursos executados diretamente pelo MS ou por transferência ao exterior, particularmente para a Organização Pan-Americana da Saúde – com redução do montante nos últimos dois anos –, e ampliação dos repasses para os municípios. Na aplicação direta, o aumento inicial se deu, especialmente a partir de 2013, com a participação federal na provisão dos profissionais no âmbito do Programa Mais Médicos, e sua queda provavelmente tem relação com a saída dos médicos cubanos em 2018 e com a substituição daquele programa pelo Médicos pelo Brasil, em 2019. Nas transferências aos municípios, houve aumento dos recursos repassados para incremento do PAB e do Teto MAC nos últimos anos por meio de emendas parlamentares ^21^ .

Em princípio, o foco na atenção básica é considerado medida crucial para a efetividade dos sistemas universais de saúde. Mas é preciso destacar que os estudos que demonstram a sua efetividade no Brasil avaliaram o modelo da Estratégia Saúde da Família (ESF) ^23^ . Não se identificaram estudos robustos sobre os efeitos do modelo “tradicional” para a saúde da população, em que o atendimento é feito estritamente na unidade básica de saúde pelos profissionais ali disponíveis. Com as medidas recentes de flexibilização dos modelos de equipes financiadas por recursos federais, a ESF passou a concorrer por recursos com o modelo tradicional ^24^^,^^25^ . Tal situação pode resultar em consequências indesejadas para a efetividade da atenção primária no país. Estimou-se que uma eventual redução da cobertura da ESF poderia elevar a taxa de mortalidade por condições sensíveis à atenção básica em 5,8% até 2030, e em 8,6% em caso de encerramento do Mais Médicos ^26^ .

Em relação aos repasses para a MAC, embora tenham aumentado na série analisada, tiveram incremento menor do que o observado para a atenção básica. Além disso, esses repasses foram mais afetados pela crise econômica e pelos contingenciamentos de pagamento de despesas do MS entre 2014 e 2016. Na aplicação direta do MS, a queda da execução a partir de 2014 pode ser explicada em parte pela diminuição dos recursos repassados aos hospitais universitários no âmbito do Programa Nacional de Reestruturação dos Hospitais Universitários Federais, que foi da ordem de R$ 630,3 milhões, em 2014, em valores constantes, e sem nenhum repasse em 2019 para o pagamento de despesas correntes. As ações e os serviços de MAC são fundamentais para assegurar a integralidade do cuidado no SUS, e estes dois níveis de complexidade são frequentemente apontados como gargalos do sistema. Assim, preocupa a baixa prioridade que tiveram nos últimos anos.

No tocante à assistência farmacêutica, a redução dos repasses está associada à decisão de centralizar a compra de medicamentos do Componente Especializado da Assistência Farmacêutica no MS, antes sob a responsabilidade dos estados. Essa decisão, em conjunto com a incorporação de novos medicamentos sob financiamento federal, incluindo vacinas, e a ampliação dos gastos com hemoderivados e judicialização explicam o crescimento expressivo da aplicação direta do MS ^27^ . Ademais, nos últimos três anos, os medicamentos do Componente Estratégico da Assistência Farmacêutica (imunobiológicos, antirretrovirais e hemoderivados) vêm ganhando espaço no gasto federal com produtos farmacêuticos, em detrimento dos itens oferecidos na atenção básica.

Quanto às despesas com vigilância em saúde, a aplicação direta do MS mais elevada em 2010 que em 2011 decorreu da implementação de ações e de serviços necessários ao enfretamento da pandemia pelo vírus *influenza* . Em 2011, o patamar de execução já era baixo e teve redução de 59% em relação a 2019. Também houve redução das despesas com gestão por aplicação direta do MS e dos repasses para projetos relacionados a essa área. No caso do MS, a queda se deu por diminuição dos gastos com pessoal ativo, que deve estar associada à redução do número de profissionais, podendo gerar maior precarização da força de trabalho no órgão.

A situação do financiamento do SUS nos últimos anos e mudanças em políticas estruturantes geram incertezas quanto ao futuro da saúde no país. Políticas de austeridade fiscal implantadas em resposta a crises econômicas têm sido apontadas como fator determinante da piora da situação de saúde da população em todo o mundo, especialmente entre os grupos socioeconomicamente mais vulneráveis ^28^^,^^29^ . No Brasil, o impacto dessas políticas sobre o SUS tem gerado preocupação quanto à possiblidade de retrocessos na oferta de bens e serviços de saúde ^30^ e no alcance: das metas relativas ao controle de doenças crônicas não transmissíveis ^31^ ; dos ODS, de forma geral ^32^ ; e da meta do ODS 3 relacionada à tuberculose ^33^ . O fato de a União ser o ente federativo com maior capacidade fiscal faz com que se espere maior esforço do governo federal no financiamento do SUS, posto que estados e municípios já se encontram no limite de suas possibilidades financeiras ^4^ . Assim, para que o Brasil alcance as metas do ODS 3, maior alocação de recursos federais se faz necessária.

Embora o cumprimento dessas metas dependa de um conjunto de ações e serviços oferecidos no âmbito de todo o sistema de saúde, e também de outras políticas públicas que atuam sobre determinantes sociais da saúde, pode-se dizer que, no que depende do SUS, esses propósitos estão mais associados a uma ou outra área finalística do sistema. As seguintes metas do ODS 3 estão bastante relacionadas à atenção básica, MAC e vigilância em saúde: 3.1 – redução da mortalidade materna; 3.2 – redução da mortalidade neonatal; 3.3 – fim das epidemias de aids, tuberculose, malária e doenças tropicais; 3.4 – redução da mortalidade prematura por doenças não transmissíveis, com promoção da saúde mental, do trabalhador e prevenção do suicídio; 3.5 – reforço à prevenção e ao tratamento decorrentes do uso de substâncias (drogas entorpecentes e uso nocivo de álcool); 3.6 – redução das mortes e lesões por acidentes no trânsito; e 3.9 – redução substancial das mortes e doenças por produtos químicos perigosos.

As seguintes metas envolvem fortemente as áreas de atenção básica, MAC e assistência farmacêutica: 3.7 – garantia de acesso universal aos serviços e insumos de saúde sexual e reprodutiva; e 3.8 – garantia de acesso a serviços essenciais de saúde de qualidade em todos os níveis de atenção e a medicamentos e vacinas incorporados ao SUS. Já estas metas se associam à vigilância em saúde e à gestão do sistema: 3a – fortalecimento da implementação da Convenção-Quadro para o Controle do Tabaco no Brasil; e 3d – reforço às capacidades locais para alerta precoce, redução e gerenciamento de emergências e riscos nacionais e globais de saúde. Por sua vez, a meta 3b – apoio à pesquisa e ao desenvolvimento de tecnologias e inovações em saúde – se associa à gestão e aos investimentos no SUS, enquanto a meta 3c – aumento substancial do financiamento da saúde e desenvolvimento de recursos humanos – envolve decisão de política fiscal que depende muito dos chefes do Poder Executivo nas três esferas de governo, no primeiro caso, e de ações no âmbito da gestão do SUS, no segundo.

Em conclusão, no caso do financiamento, houve queda no gasto per capita nos últimos anos, e as perspectivas são de redução desse indicador sob a vigência da EC 95, pelo congelamento da aplicação mínima federal no SUS e pelo atraso que causa na retomada da economia, com consequências para a arrecadação dos estados e municípios e, logo, para a destinação de recursos por esses entes à saúde ^5^^,^^6^ . Sem o aporte suficiente de recursos para o financiamento do SUS e para outras políticas que atuam sobre determinantes sociais da saúde, associado à redefinição das prioridades das políticas de saúde, o alcance das metas do ODS 3 fica comprometido, pois depende da ampliação do acesso a bens e a serviços de saúde e da melhoria da qualidade na prestação desses serviços. Caso não haja mudança no quadro atual, o risco de não cumprimento dessas metas é muito alto.
